# Case of Abdominal Tumor, Containing Hair, Teeth, &c.

**Published:** 1852-08

**Authors:** T. R. Huntington

**Affiliations:** Perry, N. Y.


ART. VI.—*Case of Abdominal Tumor, containing Hair, Teeth, etc.* By T. R. Huntington, M. D., Perry, N. Y.

The following is a brief history of a case occurring in the practice of Dr. Wells, of Moscow, by whose request I offer it to the columns of the Journal, hoping it may be of some interest to your readers. Having seen the patient but once, I am mostly indebted to Dr. Wells for its history. The patient, a female, unmarried, æt. twenty-two, was of a healthy family, herself healthy previous to the past winter, and able to perform her usual household duties until the first of March last, when she began to feel quite indisposed, but called no medical aid until the 18th, at which time Dr. W. found her suffering from a dull pain low down in the right side and in the lumbar region, the circulation very rapid, the tongue and the secretions generally natural, the urine being scanty and a little turbid, the menses had been regular and normal, the last having occurred about the first of March.

At a subsequent visit soon after, he found the abdomen somewhat enlarged, and could feel through its parietes several hard masses which were mostly confined to the iliac regions. About this time, or a little later, his attention was called to a small tumor just above the clavicle, on the outer edge of the mastoid muscle, free from pain and tenderness, but which rapidly increased in size. The size of the abdomen, also, was rapidly increased by the growth of the hardened masses, as well as by an evident effusion into the peritoneal cavity, until on the 30th of April, the distension being so great as to threaten a speedy dissolution, by the advice of Drs. McIntyre, of York, J. Huntington, of Perry, Wells and myself, a trocar was introduced and about eight pints of serous fluid allowed to escape. A large tumor then falling against the walls of the abdomen was pierced by the trocar, but its contents were of too dense a nature to pass the canula, as only a small quantity of gelatinous fluid, intermingled with caseous matter, escaped. Previous to this, different opinions were entertained by the medical advisers, in regard to the nature of the abdominal disease: some believing it to be mesenteric, but others regarding it as ovarian. All present at the operation, however, were agreed in pronouncing it ovarian. After recovering from the fatigue of the operation, the patient expressed herself somewhat relieved, but the abdomen rapidly became distended again, and the tumor in the neck so greatly increased as to occasion much difficulty in swallowing, and death supervened on the 5th of May. A post mortem examination was made on the 7th. On opening the tumor in the neck, it was found to extend as far as the upper lobe of the lung, to which it seemed to be attached, and its contents had about the consistency of softened brain, which they resembled, being mixed with an abundance of grumous blood. About twelve pints of serous fluid were then drawn from the abdominal cavity, and an incision made from the ensiform cartilage to the pubis, which exposed to view a dense, light-colored, lobulated mass, that seemed to fill nearly the entire cavity, pushing the viscera high up under the ribs, and greatly diminishing the cavity of the chest. On raising this mass is was found quite firmly adherent to the walls of the abdomen in several places, but it was attached to the left broad ligament by a flat thin neck. The uterus and right ovary were natural, but the left ovary was degenerated into this mass of disease, which, when removed, weighed eighteen pounds.

Upon an examination of the tumor, it was found to consist of many cysts, which contained a great variety of substances. Some of the larger were filled with serous or gelatinous fluid, mixed with caseous matter. Others contained an abundance of matter similar to that found in the tumor of the neck. Some of the smaller cysts contained an abundance of hair, while many others were found to contain bones and teeth.

No regularity of form or arrangement to the bones could be observed, but the teeth, of which between twenty and thirty were found, were more or less perfectly formed, and were found in groups of five or six, with their fangs impacted in bones. The walls of the cysts were in some places ossified, and cartilaginous, in others thin and membranous. Behind the peritoneum and just below the left kidney, another tumor was found, containing about a pint of matter, precisely similar to that found in the tumor of the neck, and in some of the cysts of the ovarian tumor. The lungs appeared healthy, although somewhat compressed, there being some fluid in the cavity of the chest.

